# Haikunshenxi capsule alleviates renal fibrosis in chronic kidney disease via modulation of indoxyl Sulfate–Aryl hydrocarbon receptor signaling

**DOI:** 10.3389/fphar.2026.1819011

**Published:** 2026-08-11

**Authors:** Ping Li, Chong Chen, Bangjie Zuo, Jing Zhao, Hao Tang, Baoduan Chen, Lin Qiao, Wei Sun, Sifan Sun

**Affiliations:** 1 Department of Nephrology, Affiliated Hospital of Nanjing University of Chinese Medicine, Nanjing, Jiangsu, China; 2 Department of Nephrology, Affiliated Hospital of Nantong University, Yancheng Third People’s Hospital, Yancheng, China; 3 College of Pharmacy, Nanjing University of Chinese Medicine, Nanjing, China

**Keywords:** aryl hydrocarbon receptor, chronic kidney disease, fucoidan, gut-kidney axis, Haikunshenxi capsule, indoxyl sulfate, renal fibrosis

## Abstract

**Background:**

Renal fibrosis, a common pathological endpoint in Chronic Kidney Disease (CKD), is closely associated with the accumulation of gut-derived uremic toxins, underscoring the therapeutic relevance of the gut-kidney axis. This study investigated the antifibrotic mechanism of Haikunshenxi Capsule (HKSX), a marine-derived drug, and its principal plant-derived metabolite, fucoidan (FPS), focusing on the Indoxyl Sulfate (IS)-Aryl Hydrocarbon Receptor (AhR) signaling pathway.

**Methods:**

In an adenine-induced CKD rat model, HKSX treatment significantly improved renal function and attenuated tubulointerstitial fibrosis. Targeted metabolomics analysis revealed that HKSX modulated tryptophan metabolism and reduced the systemic IS accumulation. Mechanistically, immunofluorescence and Western blotting revealed that the elevated IS levels were associated with increased *AhR* nuclear accumulation and subsequent upregulation of Cytochrome P450 enzymes (*CYP1A1/CYP1B1*) in renal tubular cells. This aberrant signaling activation was associated with Epithelial-Mesenchymal Transition (EMT), characterized by loss of E-cadherin (E-cad) and gain of α-smooth muscle actin (α-SMA).

**Results:**

HKSX treatment reduced *AhR* nuclear accumulation, downregulated CYP expression, and attenuated EMT. *In vitro* experiments using IS-stimulated HK-2 cells suggested that FPS attenuated activation of this pathway, with effects comparable to those of the specific *AhR* inhibitor CH223191 on AhR-mediated EMT-related changes.

**Conclusion:**

In conclusion, HKSX exerts a renal protective effect by reducing systemic uremic toxin accumulation and attenuating IS/AhR signaling-associated EMT, supporting the therapeutic potential of HKSX against renal fibrosis.

## 1 Introduction

As a critical global public health challenge, chronic kidney disease (CKD) is increasing in prevalence and placing a growing socioeconomic burden on societies across the world (GBD, 2025). Recent research indicates a global median CKD prevalence of 9.5% in 2023, accompanied by a median mortality rate of 2.4% (Bello et al., 2024). In China, adult prevalence has reached 8.2% (Wang et al., 2023). Driven by an aging population and the increasing incidence of comorbidities such as obesity, hypertension, and diabetes, CKD rates continue to climb (Levey and Coresh, 2012; Tong et al., 2024). Notably, approximately 11% of patients with stage 3 CKD progress to end-stage renal disease (ESRD), necessitating costly renal replacement therapies that severely impair quality of life. Current standard treatments—primarilyangiotensin-converting enzyme inhibitors (ACEIs) and angiotensin receptor blockers (ARBs)—focus on hemodynamic management but often fail to halt progression toward renal fibrosis, the common pathological endpoint of progressive chronic nephropathies (Chen et al., 2025). Several agents with anti-fibrotic potential have been investigated in recent years, including sparsentan, which has been approved for the treatment of IgA nephropathy (Colbert et al., 2025), as well as other compounds that have demonstrated renoprotective or anti-fibrotic effects in preclinical studies, such as chidamide (Liu et al., 2025). However, despite these advances, no pharmacological agent has yet been specifically approved with renal fibrosis as a primary therapeutic indication. Therefore, there is a critical demand for novel therapies that directly target the molecular drivers of renal fibrosis.

Accumulating evidence underscores the central role of gut-derived uremic toxins in CKD pathogenesis (Tang et al., 2023; Tong et al., 2024). Among these, indoxyl sulfate (IS)-a protein-bound metabolite derived from dietary tryptophan via gut microbiota metabolism and hepatic sulfation--stands out as a key contributor (Banoglu et al., 2001). IS is not merely a waste product but a potent signaling molecule whose levels correlate with renal function decline and mortality in CKD (Fujii et al., 2018). Mechanistically, IS acts as an intrinsic activator of aryl hydrocarbon receptor (AhR) in renal tubular cells, podocytes, and fibroblasts (Brito et al., 2017). Upon binding, *AhR* migrates into the nucleus, associates with the *AhR* nuclear translocator (ARNT), and triggers the transcription of cytochrome P450 enzymes, including *CYP1A1* and *CYP1B1*, via xenobiotic response elements (XRE) (Li and Fan et al., 2025; Rothhammer and Quintana, 2019; Vogel et al., 2020). In our preliminary study, sustained *AhR* activation promoted epithelial–mesenchymal transition (EMT) and fibrosis, establishing the IS/AhR signaling as a critical link between toxin accumulation and renal fibrogenesis (Chen et al., 2023). AST-120 (Kremezin), an oral indoxyl sulfate absorbent, is a therapeutic intervention to slow CKD progression, by inhibiting IS precursors binding in the gut (Andrews et al., 2025; Hsu et al., 2022). However, the benefits are limited (Lee et al., 2024; Schulman et al., 2015). AST-120 does not directly inhibit activated *AhR* signaling in kidney tissues, and its long-term application is constrained by gastrointestinal side effects and uncertain effects on disease progression. These limitations highlight the need for novel specifically targeted agents that can reduce IS accumulation.

Marine-derived natural products have attracted growing interest for their distinctive chemical profiles and bioactivities (Li and She et al., 2025; Ma et al., 2025; Zhou et al., 2018). Fucoidan (FPS), a fucose-rich sulfated polysaccharide derived from brown algae, shows diverse pharmacological properties, including antioxidative, anti-inflammatory, and renoprotective actions (Wang et al., 2021; Zahan et al., 2022). Clinically, Haikunshenxi (HKSX) capsule, a formulated preparation centered on fucoidan, has been used in CKD and has been shown to reduce the concentrations of serum creatinine and blood urea nitrogen (Wang et al., 2019). Despite its potential in alleviating renal fibrosis, the underlying mechanisms, particularly whether it interferes with the IS/AhR signaling pathway, remain unclear.

Based on this background, we hypothesize that the renoprotective effects of HKSX and its principal plant-derived metabolite fucoidan involve modulation of IS/AhR signaling. This study aims to investigate whether HKSX could suppress IS accumulation and attenuate IS-induced *AhR* activation, downregulating *CYP1A1/CYP1B1* expression, thereby inhibiting EMT and reducing ECM deposition.With combined *in vivo* and *in vitro* approaches, we seek to provide a mechanistic foundation for the use of HKSX in the treatment of renal fibrosis.

## 2 Materials and methods

The manuscript was reviewed using the GA-online best practice tool for ethnopharmacology reporting standards. Compliance with the GA-online guidelines has been summarized in Supplementary Tables S1, S2a, which are provided as Supplementary Material (PDF format) for reference.

### 2.1 Chemicals and reagents

Botanical authentication. The fucoidan (FPS) used in this study is derived from *Saccharina japonica* (J.E. Areschoug) C.E. Lane, C. Mayes, Druehl & G.W. Saunders [Laminariaceae; Laminariae Thallus]. The species name was validated using the AlgaeBase database (https://www.algaebase.org).

Haikunshenxi Capsule and fucoidan. HKSX is a marketed standardized marine-derived fucoidan-based botanical drug preparation approved by the National Medical Products Administration (NMPA) of China (Approval No.: Z20030052; Manufacturer: Jilin Huinan Changlong Biochemical Pharmaceutical Co., Ltd., Jilin, China). The product complies with the China National Drug Standard YBZ00892003-2006. Each capsule contains 0.22 g of total material. The capsule contents were opened, pooled, and the required amount of powder was suspended in 0.5% sodium carboxymethyl cellulose (CMC-Na) solution for oral administration to rats.

The FPS used for *in vitro* experiments was provided by the same manufacturer and was from the same batch (Batch No. 015240601; production date: 1 June 2024; expiry date: 31 May 2026). For cell culture, FPS was dissolved in sterile phosphate-buffered saline (PBS) or culture medium, and filtered through a 0.22 µm filter before use.

As the principal plant-derived metabolite of a nationally approved drug, the FPS used in this study conforms to the China National Drug Standard YBZ00892003-2006. This standard defines the key quality characteristics of fucoidan from S. japonica, including a total polysaccharide content of ≥25.0% and a sulfate group content of ≥22.0%, with fucose as the major monosaccharide [2†L12-L15]. No further independent chemical characterization was performed, as the material is a commercial pharmaceutical plant-derived metabolite with a legally enforceable quality standard.

Human Kidney-2 (HK-2) cells (CC4008) were purchased from Guangzhou SAIKU Biotechnology Co., Ltd. (Guangzhou, China).

Other reagents. Adenine (A8626) and Indoxyl Sulfate (IS, I3875) were sourced from Sigma-Aldrich (St. Louis, MO, USA). AST-120 (Kremezin) was obtained from Kureha Chemical Industry (Tokyo, Japan). CH-223191 (HY-12684) was purchased from MCE (Monmouth Junction, NJ, USA). Regarding antibodies, the anti-AhR polyclonal antibody (AF01491) was purchased from AiFang (Hunan, China), and the anti-COL1 antibody (ab260043) was obtained from Abcam (Cambridge, UK). Antibodies against α-smooth muscle actin (α-SMA, 14395-1-AP), E-cadherin (20874-1-AP), *CYP1A1* (13241-1-AP), and *CYP1B1* (18505-1-AP) were all acquired from Proteintech (Wuhan, China).

### 2.2 Animals and experimental design

Thirty male Sprague-Dawley (SD) rats, 8 weeks old and weighing 180–220 g, free of specific pathogens (SPF), were obtained from Beijing Vital River Laboratory Animal Technology Co., Ltd. (Beijing, China). The animals were maintained under controlled conditions, with a temperature of 22 °C ± 2 °C, relative humidity of 50%–60%, and a 12-h light/dark cycle, and had free access to food and water. All procedures were approved by the Animal Ethics Committee of the Affiliated Hospital of Nanjing University of Chinese Medicine (Approval No. 2024DW-058-01).

Following a 1-week acclimation period, the rats were randomly assigned to five groups (n = 6 per group). Dosages were determined according to the body surface area conversion between humans and rats. The experimental groups were as follows: Control Group: Rats were administered 0.9% saline *via* gastric gavage for 8 weeks. Model Group (Adenine): Rats were administered a 2.5% adenine suspension (200 mg/kg/day) *via* gastric gavage for 8 weeks. HKSX-L Group: Rats were administered adenine (200 mg/kg/day) together with a low dose of HKSX suspension (0.0693 g/kg/day) for 8 weeks. HKSX-H Group: Rats were administered adenine (200 mg/kg/day) together with a high dose of HKSX suspension (0.1386 g/kg/day) for 8 weeks. AST-120 Group: Rats were administered adenine (200 mg/kg/day) together with AST-120 (4 g/kg/day) for 8 weeks.

### 2.3 Sample collection

At the end of the 8-week intervention, rats were individually placed in metabolic cages to obtain 24-h urine samples. Subsequently, rats were first anesthetized with isoflurane, followed by collection of blood from the abdominal aorta, and the rats were sacrificed. Bilateral kidneys were excised, the fascia was removed, and the kidneys were weighed. A portion of the left kidney (sagittal section from the same site) was fixed in 4% paraformaldehyde at 4 °C for histological analysis. The leftover kidney tissues were promptly frozen in liquid nitrogen and kept at −80 °C for subsequent analyses.

### 2.4 Cell culture and treatment

Human renal proximal tubular epithelial cells (HK-2) were cultured in DMEM/F12 medium supplemented with 10% fetal bovine serum (FBS) at 37 °C in a humidified incubator with 5% CO_2_. Purified FPS was used instead of HKSX-containing serum to minimize interference from serum-derived components and to specifically assess the effect of FPS on IS-induced *AhR* signaling under controlled conditions. Cells were randomly divided into four groups: Blank Control Group, cultured in normal medium; IS Group, treated with 1 mmol/L Indoxyl Sulfate; IS + FPS Group, treated with 1 mmol/L IS and 50 ug/mL FPS; and CH223191 Group, treated with 1 mmol/L IS and 5 μmol/L CH223191 (an *AhR* inhibitor). All treatments were maintained for 24 h.

### 2.5 Assessment of renal and liver function

Serum levels of creatinine (Scr), blood urea nitrogen (BUN), aspartate aminotransferase (AST), and alanine aminotransferase (ALT), as well as urine total protein (24 h-UTP) were measured using an automatic biochemical analyzer.

### 2.6 Histopathological and immunohistochemical (IHC) analysis

Kidneys fixed in 4% paraformaldehyde were paraffin-embedded and sectioned. Sections underwent deparaffinization, rehydration, and staining with H&E or Masson’s trichrome to examine morphology and fibrosis, with images taken under a microscope.

For IHC, sections were retrieved in citrate buffer and exposed to 3% hydrogen peroxide to eliminate endogenous peroxidase activity. After blocking with avidin/biotin and streptavidin, sections were incubated overnight at 4 °C with primary antibodies against *AhR* and COL-1. Sections were subsequently incubated with a goat anti-rabbit secondary antibody for 1 h, followed by color development using diaminobenzidine (DAB). The proportion of positively stained areas was quantified with ImageJ software.

### 2.7 Immunofluorescence staining

Kidney sections embedded in paraffin were deparaffinized using xylene and gradually rehydrated through a series of ethanol concentrations. Antigen retrieval was performed by microwave heating in sodium citrate buffer (10 mM, pH 6.0). After washing with PBS, sections were permeabilized with 0.3% Triton X-100 for 15 min and blocked with 10% normal goat serum for 1 h at room temperature. The sections were incubated overnight at 4 °C with primary antibodies against COL-1, *CYP1A1*, and *AhR*. Following washing, sections were incubated with appropriate Alexa Fluor-Conjugated secondary antibodies (Invitrogen, 1:500) for 1 h at 37 °C in the dark. Nuclei were counterstained with 4′,6-diamidino-2-phenylindole (DAPI). Images were acquired using a Confocal laser scanning microscope (Leica TCS SP8, Leica Microsystems, Wetzlar, Germany). Fluorescence intensity was quantified using ImageJ software.

### 2.8 Western blot analysis

Total protein was extracted from kidney tissues and HK-2 cells using lysis buffer. The protein concentration was determined using a BCA Protein Assay Kit. Samples were mixed with loading buffer and denatured at 100 °C. Proteins were resolved on 10% SDS-PAGE gels and transferred to PVDF membranes. Following blocking, membranes were incubated with primary antibodies overnight at 4 °C. The membranes were then washed with TBST and incubated with HRP-Conjugated goat anti-rabbit or goat anti-rat IgG (1:10,000) secondary antibodies. Protein bands were visualized using an enhanced chemiluminescence (ECL) system and analyzed using ImageJ.

### 2.9 Quantitative real-time PCR (RT-qPCR)

Total RNA was extracted from kidney tissues and HK-2 cells using an RNA extraction kit. RNA Concentration was measured, and reverse transcription was performed to synthesize cDNA using a Reverse Transcription Kit (42 °C for 15 min, 95 °C for 3 min). RT-qPCR was performed using a fluorescence quantitative PCR instrument. PCR was performed under the following cycling conditions: 95 °C for 15 min for pre-denaturation, then 40 cycles of 95 °C for 10 s and 65 °C for 30 s for denaturation and annealing/extension, respectively. Relative expression of mRNA was calculated using the 2^-ΔΔCt method, with β-actin serving as the internal control. Primers were synthesized by GenScript Biotech, and the sequences are listed as follows: COL-1(Rat) forward: 5’-AGC​CCA​AAC​CCC​AAG​GAG-3’; and reverse: 5’-CAT​CGG​CAG​GAT​CGG​AAC-3’; α-SMA (Rat) forward: 5’-AGG​AAT​ACG​ATG​AAG​GGG​GG-3’ and reverse: 5’TGC​TAG​AGA​CAG​AGA​GGA​GCA-3’; *AhR* (Rat) forward: 5’- AGTTGTGTCAGGTGGAGG -3’ and reverse: 5’- GGAGCAAAGTTCTGTGTG -3’; *CYP1A1* (Rat) forward: 5’- ATCCTTTGTCCCATTCAC -3’ and reverse: 5’- GATCACCCCATAGTTCCT -3’; CYP1B1 (Rat) forward: 5’- CCCCAAAAACACGGTAGT -3’ and reverse: 5’- GAGCGAGGATGGAGATGA -3’; β-actin (Rat) forward: 5’-GAG​AGG​GAA​ATC​GTG​CGT-3’ and reverse: 5’-GGA​GGA​AGA​GGA​TGC​GG-3’; COL-1(Homo) forward: 5’-TGG​CAA​AGA​AGG​CGG​CAA​AGG-3’; and reverse: 5’-AGG​AGC​ACC​AGC​AGG​ACC​ATC-3’; α-SMA (Homo) forward: 5’-AGG​AAT​ACG​ATG​AAG​GGG​GG-3’ and reverse: 5’TGC​TAG​AGA​CAG​AGA​GGA​GCA-3’; *AhR* (Homo) forward: 5’- ACA​ACC​GAT​GGA​CTT​GGG​TC -3’ and reverse: 5’- TGG​CAG​GAA​AAG​GGT​TGG​TT -3’; *CYP1A1* (Homo) forward: 5’- AAT​TTC​GGG​GAG​GTG​GTT​GG -3’ and reverse: 5’- AGG​CAT​TCA​GGG​AAG​GGT​TG-3’; *CYP1B1* (Homo) forward: 5’- ACG​CCT​TTA​TCC​TCT​CTG​CG -3’ and reverse: 5’- GTG​ATA​GTG​GCC​GGT​ACG​TT -3’; β-actin (Homo) forward: 5’-CTA​CCT​CAT​GAA​GAT​CCT​CAC​CGA-3’ and reverse: 5’-TTC​TCC​TTA​ATG​TCA​CGC​ACG​ATT​T-3’.

### 2.10 Targeted metabolomics analysis (HPLC-MS/MS)

Sample Preparation: A 50 μL serum sample was extracted with 250 μL of methanol containing internal standards (250 ng/mL). After vortexing the mixture for 3 min, it was incubated at −20 °C for 30 min and centrifuged at 12,000 rpm for 10 min at 4 °C. A 150 μL aliquot of the supernatant was then collected and subjected to a second centrifugation at 12,000 rpm for 5 min at 4 °C. Ultimately, 100 μL of the supernatant was taken for LC-MS/MS analysis.

LC-MS/MS Conditions: Analysis was performed using a UPLC system coupled with a QTRAP® 6500+ Mass Spectrometer. Chromatographic separation was performed using a Waters ACQUITY UPLC HSS T3 C18 column (100 mm × 2.1 mm i. d., 1.8 µm) at 40 °C. The mobile phases were composed of water containing 0.1% formic acid (A) and acetonitrile containing 0.1% formic acid (B). The gradient elution program was: 0–1 min, 10% B; 1–6 min, 10%–95% B; 6–7 min, 95% B; 7.1–10 min, 10% B. The system was operated at a flow rate of 0.35 mL/min, with an injection volume of 2 μL. Mass Spectrometry: The system operated in both positive and negative ion modes using an ESI Turbo Ion-Spray interface. Parameters were set as follows: source temperature, 550 °C; ion spray voltage, 5500 V (positive)/−4500 V (negative); curtain gas, 35 psi. Tryptophan and its metabolites were analyzed using scheduled multiple reaction monitoring (MRM). Data acquisition and quantification were performed using Analyst 1.6.3 and MultiQuant 3.0.3 software (Sciex), respectively.

### 2.11 Statistical analysis

Data are expressed as mean ± standard error of the mean (SEM). All statistical analyses were performed with GraphPad Prism software. One-way ANOVA followed by Tukey’s *post hoc* test was used to evaluate differences between groups. P-values below 0.05 were regarded as statistically significant.

## 3 Results

### 3.1 Targeted metabolomic profiling of tryptophan pathway in serum from adenine-induced CKD rats by LC-MS/MS

Based on our preliminary the untargeted metabolomics findings, which indicated pronounced disruptions in amino acid metabolism (Chen et al., 2023), this study focused specifically on the tryptophan metabolic pathway for targeted investigation. We performed quantitative metabolomic profiling of serum from the control (Con group) and adenine-induced CKD rats (Ade group) using liquid chromatography-tandem mass spectrometry (LC-MS/MS).

**FIGURE 1:**
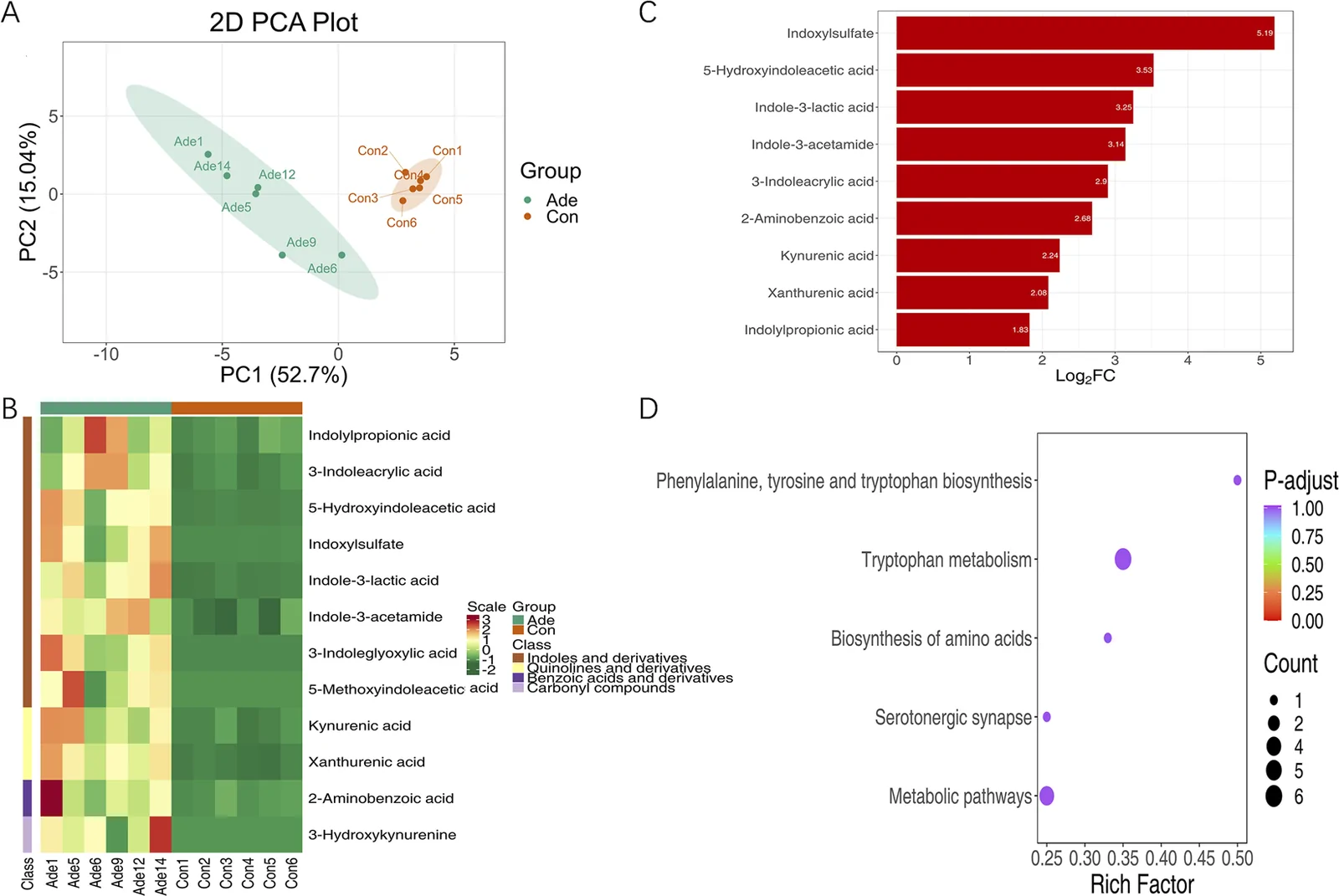
Targeted metabolomics analysis reveals tryptophan metabolism dysregulation in adenine-induced CKD rats. **(A)** Principal component analysis (PCA) score plot based on targeted tryptophan metabolomic profiling of serum samples from Control (Con) and adenine-treated (Ade) rats, showing a clear separation between the two groups. **(B)** Heatmap analysis illustrating the relative levels of tryptophan metabolism-related metabolites in rat serum. Each row represents an individual metabolite, and each column represents a sample. **(C)** Quantitative analysis of significantly altered metabolites involved in the tryptophan metabolic pathway in the group treated with Ade versus the Control group. **(D)** Pathway enrichment analysis using KEGG for differential metabolites, revealing pathways significantly associated with tryptophan metabolism.

Principal Component Analysis (PCA) of the metabolic data revealed a clear separation between the two groups (Figure 1A). The Ade group clustered distinctly from the Con group along the first principal component (PC1, 52.7% variance explained), indicating a substantial adenine-induced shift in the systemic metabolic profile.

To identify metabolites responsible for this separation, we analyzed the differential expression patterns. The fold-change (FC) analysis (Figure 1B) highlighted significant alterations in metabolite levels, with red and green bars denoting up- and downregulated metabolites, respectively. Consistently, a hierarchical clustering heatmap (Figure 1C) displayed group-specific metabolic signatures, showing a cluster of markedly upregulated metabolites (deep red) in the Ade group compared to controls. Importantly, this analysis demonstrated accumulation of IS and other tryptophan-derived uremic toxins, along with decreased levels of tryptophan and its beneficial metabolites.

Enrichment analysis of pathways using the Kyoto Encyclopedia of Genes and Genomes (KEGG) database (Figure 1D). further elucidated the biological relevance of these changes. “Tryptophan metabolism” was identified as one of the pathways most significantly enriched. (high Rich factor and low p-value). Together, these metabolomic findings suggest that the adenine-induced CKD model successfully recapitulates uremia-like metabolic dysregulation, particularly along the tryptophan-indole metabolic axis.

### 3.2 HKSX treatment improves renal function and attenuates histological damage in adenine-induced CKD rats

**FIGURE 2:**
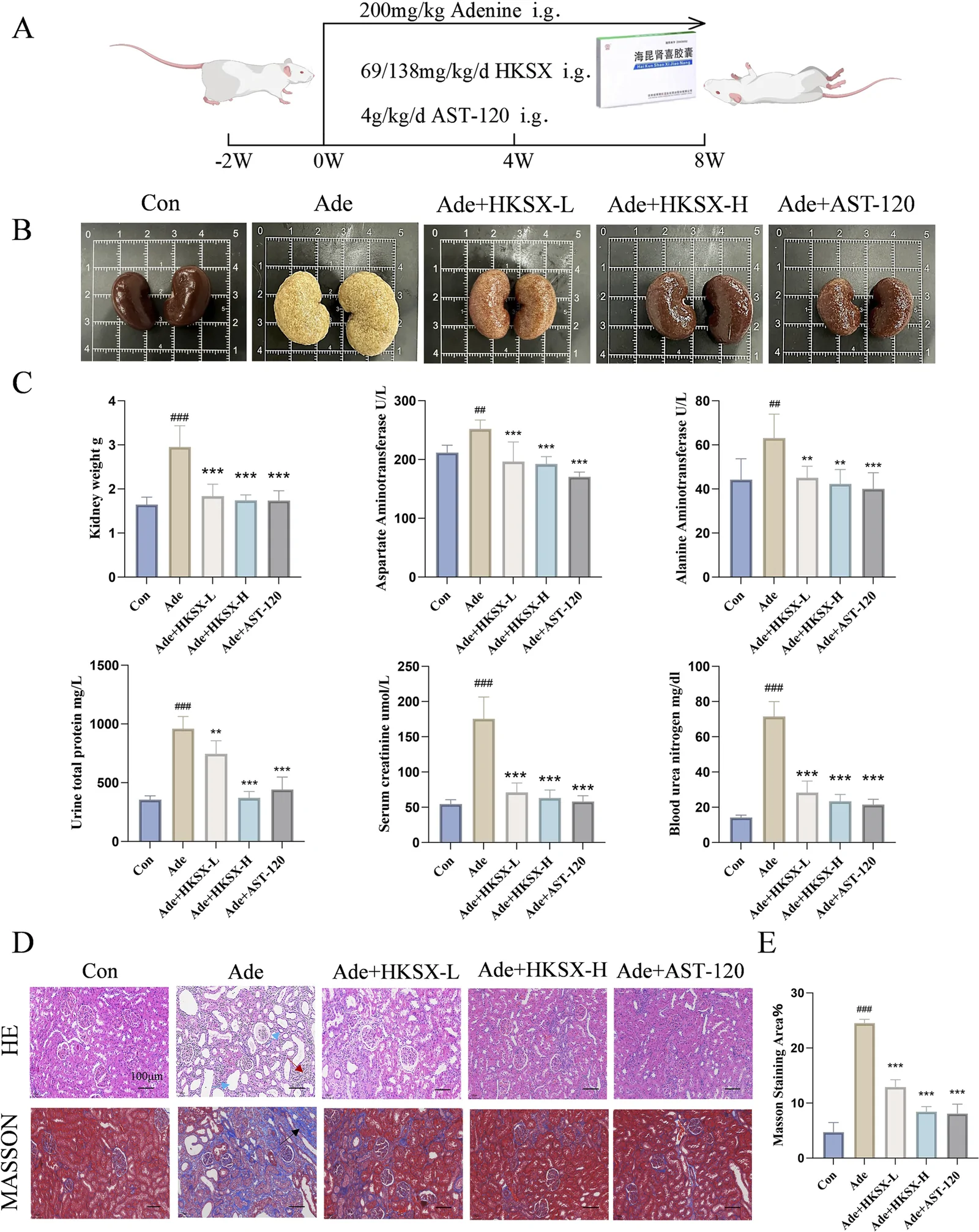
Evaluation of the protective effects of HKSX on renal function and histopathology. **(A)** Schematic diagram of the experimental protocol and treatment schedule (n = 6). **(B)** Representative gross morphological images of kidneys from each group. **(C)** Biochemical analysis including kidney weight, liver function markers (AST, ALT), and renal function markers (BUN, UTP, Scr). **(D)** Representative photomicrographs of kidney tissues stained with Hematoxylin and Eosin (H&E) for structural assessment and Masson’s trichrome for fibrosis visualization. Red arrows indicate inflammatory cell infiltration; blue indicate tubular atrophy and tubular dilation; black arrows indicate collagen deposition. (Scale bar = 100 μm, n = 6). **(E)** Quantitative analysis of the Masson-positive fibrotic area percentage. Data are expressed as mean ± SEM. ^#^ *p* < 0.05, ^##^ *p* < 0.01, ^###^ *p* < 0.001 versus the Control group; ^*^ *p* < 0.05, ^**^ *p* < 0.01, ^***^ *p* < 0.001 versus the Model group.

HKSX treatment improved renal function and attenuated histopathological damage in an adenine-induced CKD rat model. Following the experimental design (Figure 2A), we first evaluated general health and renal function markers. Gross macroscopical examination (Figure 2B) showed that kidneys from the Model group were noticeably enlarged, pale, and exhibited granulated surfaces, suggesting substantial tissue injury and hypertrophy. Consistently, kidney weight was significantly increased in the Model group (Figure 2C). HKSX administration effectively ameliorated these macroscopic abnormalities and reduced renal hypertrophy. As shown in Figure 2C, adenine administration induced severe renal impairment, as demonstrated by significant elevations in urinary total protein (UTP), serum creatinine (Scr), and blood urea nitrogen (BUN) compared with the Control group. However, HKSX treatment dose-dependently reduced these elevations, indicating restored renal filtration function. Notably, no significant changes in aspartate aminotransferase (AST) and alanine aminotransferase (ALT) levels were observed among the groups (Figure 2C), indicating that HKSX did not cause detectable hepatotoxicity at the administered doses.

Histopathological analysis supported these findings. Hematoxylin and eosin (H&E) staining (Figure 2D) revealed widespread tubular atrophy, dilation, and infiltration of inflammatory cells in the Model group. Masson’s trichrome staining confirmed extensive collagen deposition in the tubulointerstitium, indicating severe fibrosis. Quantitative assessment (Figure 2E) showed a marked increase in interstitial fibrosis in the Model group, which was significantly attenuated by HKSX treatment.

Together, these results suggest that HKSX effectively protects against functional deterioration and structural remodeling in adenine-induced CKD rats.

### 3.3 HKSX alleviates renal fibrosis in adenine-induced CKD rats

**FIGURE 3:**
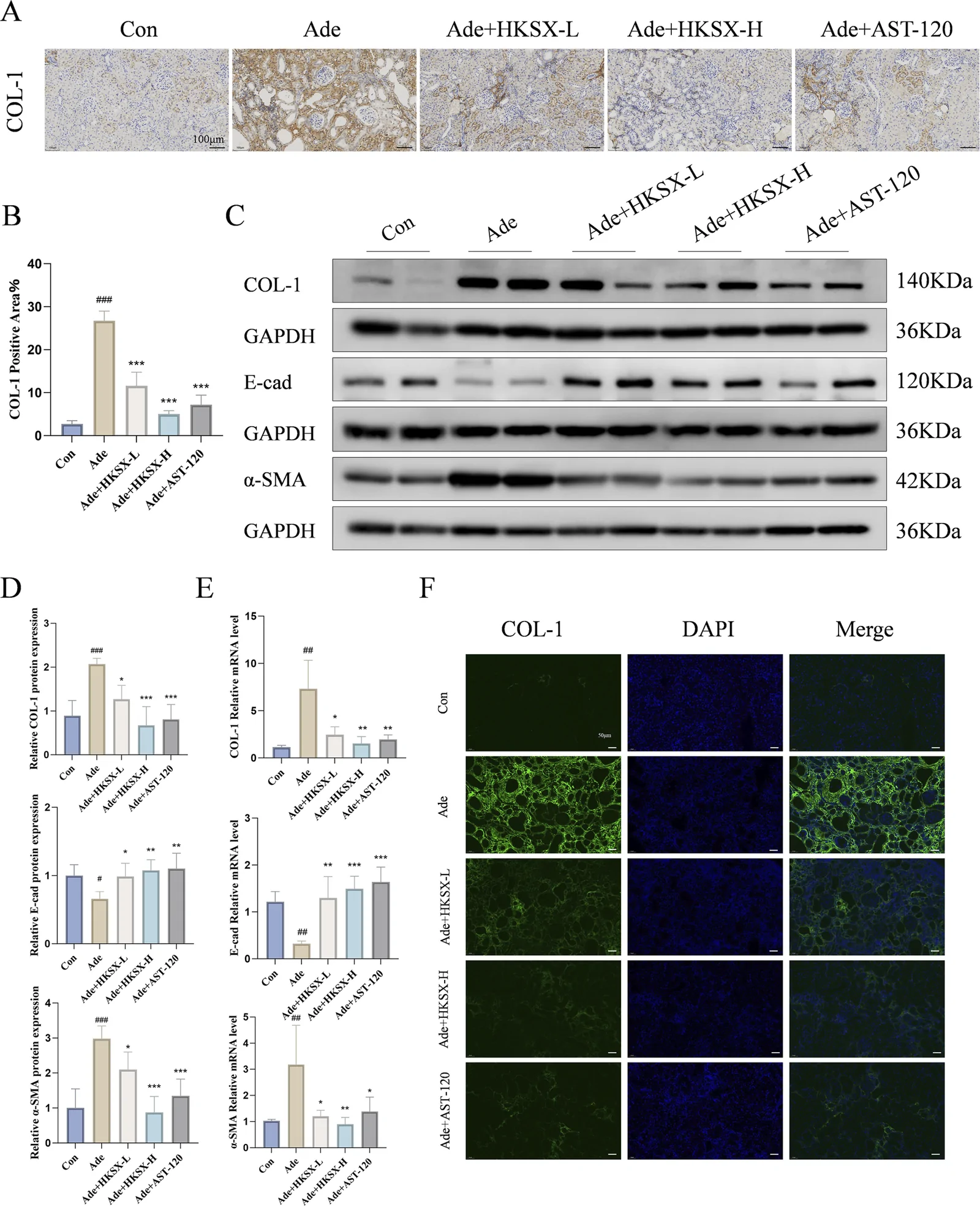
HKSX attenuates renal fibrosis and inhibits EMT. **(A)** Representative immunohistochemical images of COL-1 in kidney tissues from all groups at the conclusion of the experiment (scale bar = 100 μm, n = 5). **(B)** Quantitative analysis of the COL-1 immunohistochemistry-positive area in kidney tissues from each group. **(C)** Western blot results showing the protein expression levels of COL-1, E-cad, and α-SMA in kidney tissues from each group (n = 6). **(D)** Quantitative analysis of the relative protein expression levels of COL-1, E− Cad, and α-SMA in kidney tissues from each group (n = 6). **(E)** Real-time quantitative PCR analysis of mRNA expression levels of COL-1, E− Cad, and α-SMA in kidney tissues from each group (n = 6). **(F)** Representative immunofluorescence staining images of COL-1 (Scale bar = 50 μm, n = 5). Data are expressed as mean ± SEM. ^#*p*^ < 0.05, ^##*p*^ < 0.01, ^###*p*^ < 0.001 versus the Control group; ^*p^ < 0.05, ^**p^ < 0.01, ^***p^ < 0.001 versus the Model group.

To systematically evaluate the antifibrotic efficacy of HKSX, we assessed the expression of Collagen Type I (COL-1), a major extracellular matrix component. Immunohistochemical (IHC) staining revealed substantial COL-1 deposition in renal tissues of the Model group, with intense brownish-yellow positive signals localized predominantly in the tubulointerstitial area, confirmed by the quantitative analysis and immunofluorescence (IF) staining (Figures 3A,B,F). However, HKSX treatment remarkably attenuated both the intensity and distribution of COL-1 staining in IHC and IF assays, suggesting its potent suppressive effect on matrix deposition.

We further explored the molecular mechanism contributing to this structural improvement. Western blot analysis of renal tissues revealed distinct phenotypic alterations indicative of Epithelial-Mesenchymal Transition (EMT) in the Model group (Figure 3C). These alterations involved marked decreases in the epithelial marker E-cadherin, accompanied by strong increases in mesenchymal and fibrotic markers, such as α-SMA and COL-1 (Figure 3C). Quantitative analysis further confirmed these expression changes (Figure 3D).

RT-qPCR analysis confirmed parallel transcriptional changes, with marked upregulation of COL-1 and α-SMA mRNA and suppression of E-cadherin (E-Cad) in the Model group. (Figure 3E). HKSX administration effectively reversed these pathological alterations, restoring E-cad expression and significantly suppressing both protein and mRNA expression of α-SMA and COL-1.

Together, these findings suggest that HKSX attenuates renal fibrosis, accompanied by reduced EMT-related changes and extracellular matrix component expression.

### 3.4 HKSX modulates the activation of IS/AhR signaling pathway in adenine-induced CKD rats

**FIGURE 4:**
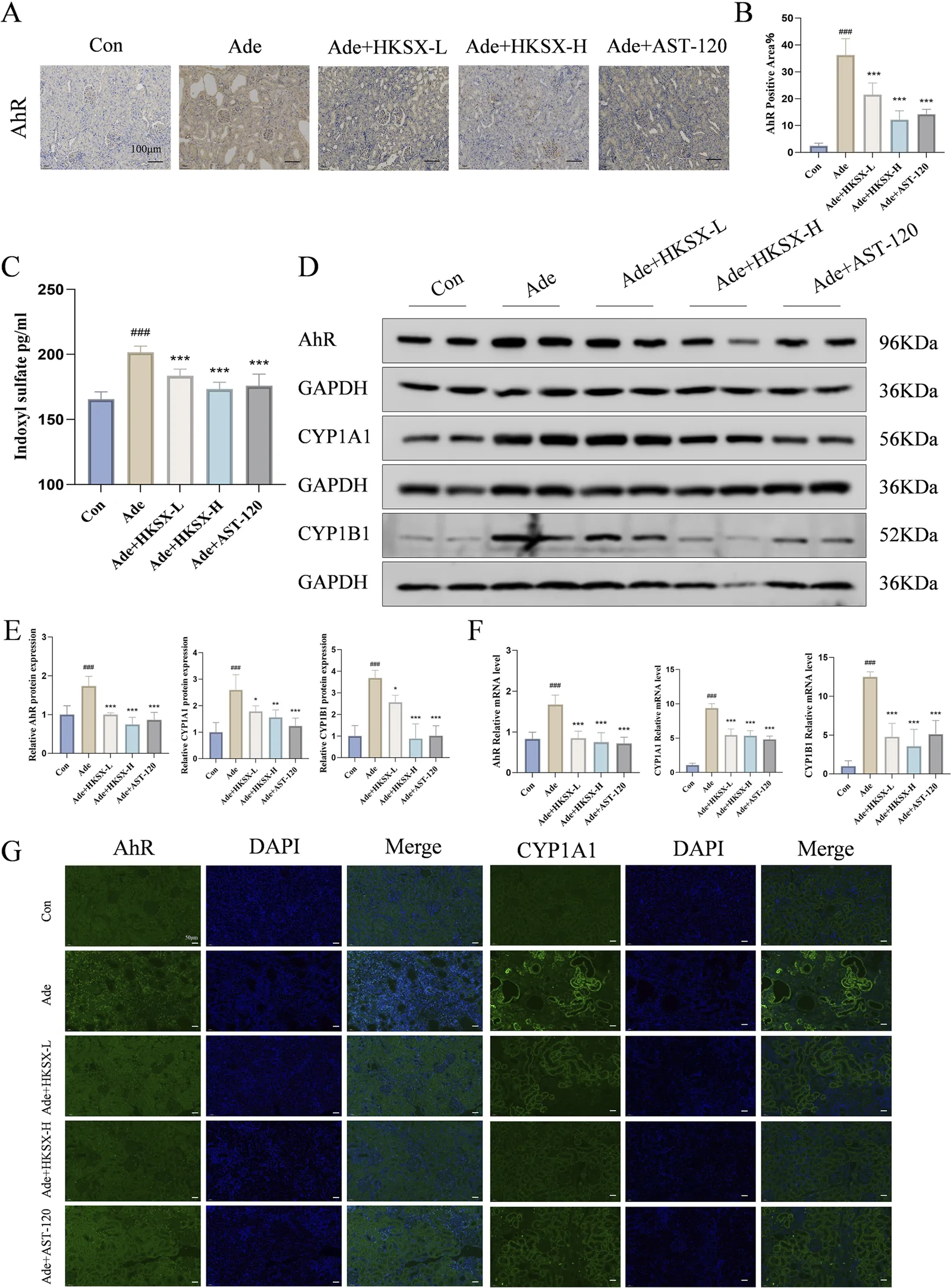
HKSX inhibits the IS/AhR signaling axis in adenine-induced CKD rats. **(A)** Representative IHC images of *AhR* in kidney tissues from all groups at the conclusion of the experiment (scale bar = 100 μm, n = 5). **(B)** Quantitative analysis of the percentage of *AhR* positive staining area. **(C)** Determination of serum IS levels in SD rats. **(D)** Representative Western blot bands showing the protein expression of *AhR*, *CYP1A1*, and *CYP1B1*. *GAPDH* served as the internal loading Control. **(E)** Densitometric analysis of the relative protein expression of *AhR*, *CYP1A1*, and *CYP1B1*. **(F)** Relative mRNA expression levels of *AhR*, *CYP1A1*, and *CYP1B1* determined by RT-qPCR. **(G)** Representative immunofluorescence staining images of *AhR* (green) and *CYP1A1* (green) (Scale bar = 50 μm, n = 5). Arrows indicate increased nuclear accumulation of *AhR*. Data are expressed as mean ± SEM. ^#^ *p* < 0.05, ^##^ *p* < 0.01, ^###^ *p* < 0.001 versus the Control group; ^*^ *p* < 0.05, ^**^ *p* < 0.01, ^***^ *p* < 0.001 versus the Model group.

Given that IS is a potent endogenous ligand of *AhR*, we first examined whether HKSX affects systemic levels of this uremic toxin. As shown in Figure 4C, the serum concentration of IS was significantly higher in the Adenine-induced Model group than in the Control group. HKSX administration markedly decreased serum IS levels in a manner dependent on the dose, suggesting its ability to target the gut-kidney axis and attenuate the source of profibrotic stimuli.

To assess whether elevated IS activates downstream signaling in renal tissues, we evaluated the activation status of *AhR* and its target enzymes, *CYP1A1* and *CYP1B1*. Immunohistochemical (IHC) staining showed a marked increase in *AhR* positive staining in renal tubules of Model rats (Figures 4A,B). Importantly, immunofluorescence (IF) staining (Figure 4G) provided visual evidence consistent with *AhR* activation: while *AhR* was predominantly localized in the cytoplasm in the Control animals, clear nuclear accumulation was observed in the Model group, accompanied by strong fluorescent expression of the downstream enzyme *CYP1A1*.

These findings were further confirmed at the protein and transcriptional levels. Western blot analysis revealed robust upregulation of *AhR*, *CYP1A1*, and *CYP1B1* protein expression in the Model group (Figures 4D,E). Consistently, RT-qPCR results showed a significant elevated mRNA expression of *AhR*, *CYP1A1*, and *CYP1B1* (Figure 4F). HKSX administration effectively reversed these alterations, reducing *AhR* nuclear accumulation, downregulating AhR and CYP protein expression, and suppressing their transcriptional levels.

Collectively, these results suggest that HKSX may exert renal protection, which is associated with reduced systemic IS burden and attenuated IS/AhR signaling activation.

### 3.5 FPS ameliorates fibrosis by inhibiting the IS/AhR signaling pathway in HK-2 cells

To investigate the molecular mechanism underlying the protective effect of FPS, with an emphasis on the function of *AhR*, we performed *in vitro* experiments using HK-2 cells. Additional CCK-8 cell viability data and FPS-only Western blot results are provided in Supplementary Figure S1, confirming that FPS alone does not affect normal HK-2 cells. The specific *AhR* inhibitor, CH223191, was employed as a positive control to validate *AhR* signaling dependency.

**FIGURE 5:**
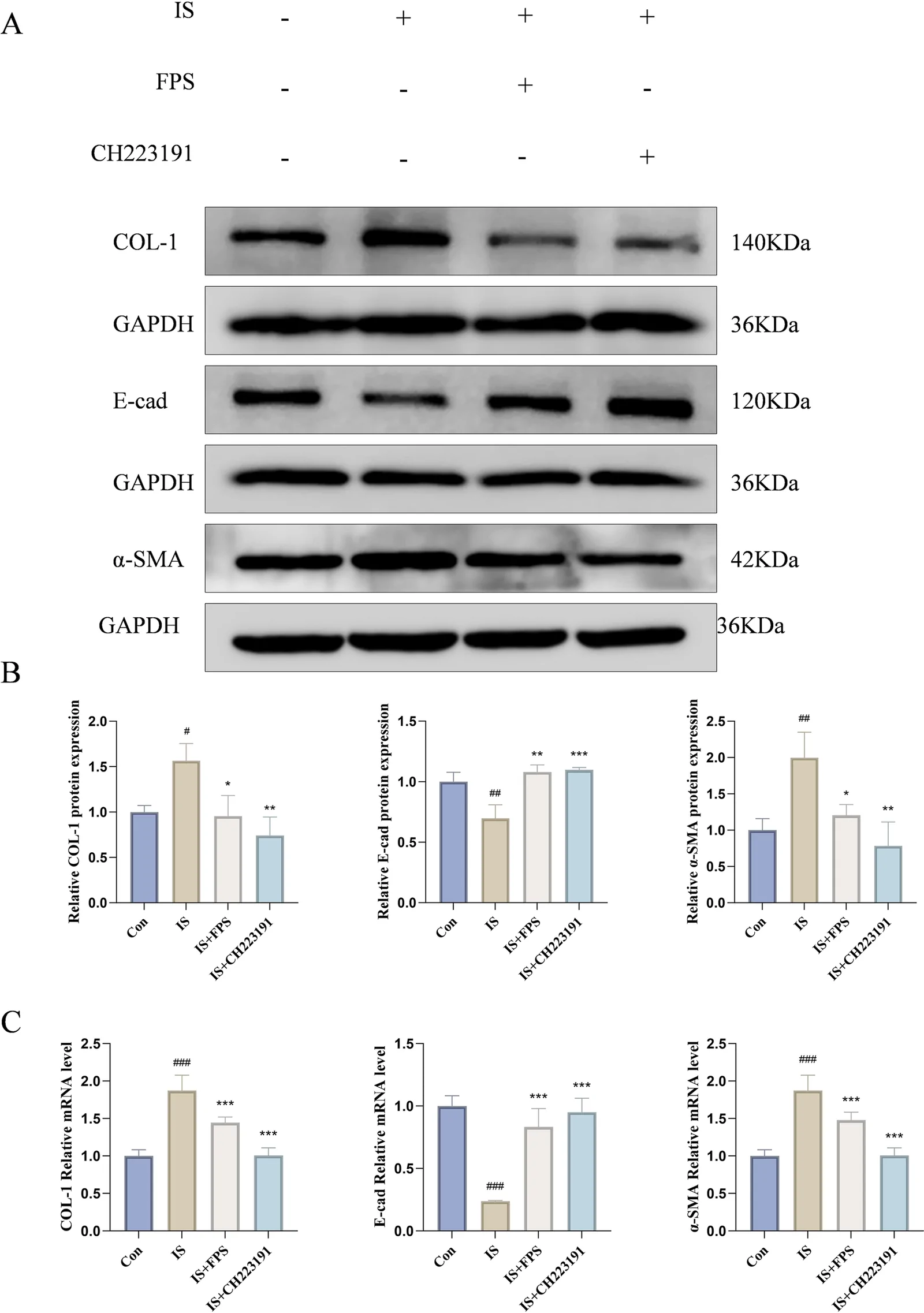
FPS and CH223191 attenuate IS-mediated EMT in HK-2 cells. **(A)** Representative Western blot bands showing the protein expression of COL-1, E-cad, and α-SMA in HK-2 cells exposed to IS, FPS, or CH223191. *GAPDH* served as the internal loading Control. **(B)** Densitometric analysis of the relative protein expression levels. **(C)** Relative mRNA expression levels of COL-1, E-cad, and α-SMA determined by RT-qPCR. Data are presented as mean ± SEM (n = 3). Data are expressed as mean ± SEM. ^#^ *p* < 0.05, ^##^ *p* < 0.01, ^###^ *p* < 0.001 versus the Control group; ^*^ *p* < 0.05, ^**^ *p* < 0.01, ^***^ *p* < 0.001 versus the Model group.

Western blot analysis revealed that stimulation with IS induced a pronounced EMT, characterized by significant downregulation of the epithelial marker E-cad and concurrent upregulation of the mesenchymal/fibrotic markers α-SMA and COL-1 (Figures 5A,B). Pretreatment with CH223191 effectively blocked these IS-induced changes, restoring E-cad expression and suppressing α-SMA and COL-1 levels, supporting that IS-induced EMT-related changes are dependent on *AhR* activation.

Notably, treatment with FPS exhibited protective effects comparable to those of CH223191. FPS significantly reversed IS-induced alterations in the protein-level expression of E-cad, α-SMA, and COL-1. RT-qPCR analysis (Figure 5C) further confirmed these findings transcriptionally: both FPS and CH223191 markedly attenuated IS-mediated upregulation of COL-1 and α-SMA mRNA while restoring E-cad expression. Taken together, these results suggest that FPS showed effects comparable to those of an *AhR* inhibitor, attenuating IS-induced EMT-related changes associated with *AhR* signaling activation.

### 3.6 FPS inhibits IS/AhR signaling pathway activation in HK-2 cells

Given that IS-induced EMT-related changes are dependent on *AhR* activation, we further investigated whether the marine sulfated polysaccharide FPS modulates this upstream signaling cascade. We assessed *AhR* expression and its downstream metabolic targets, *CYP1A1* and *CYP1B1*, in HK-2 cells, using the selective *AhR* inhibitor, CH223191, as a positive control to validate pathway inhibition.

**FIGURE 6:**
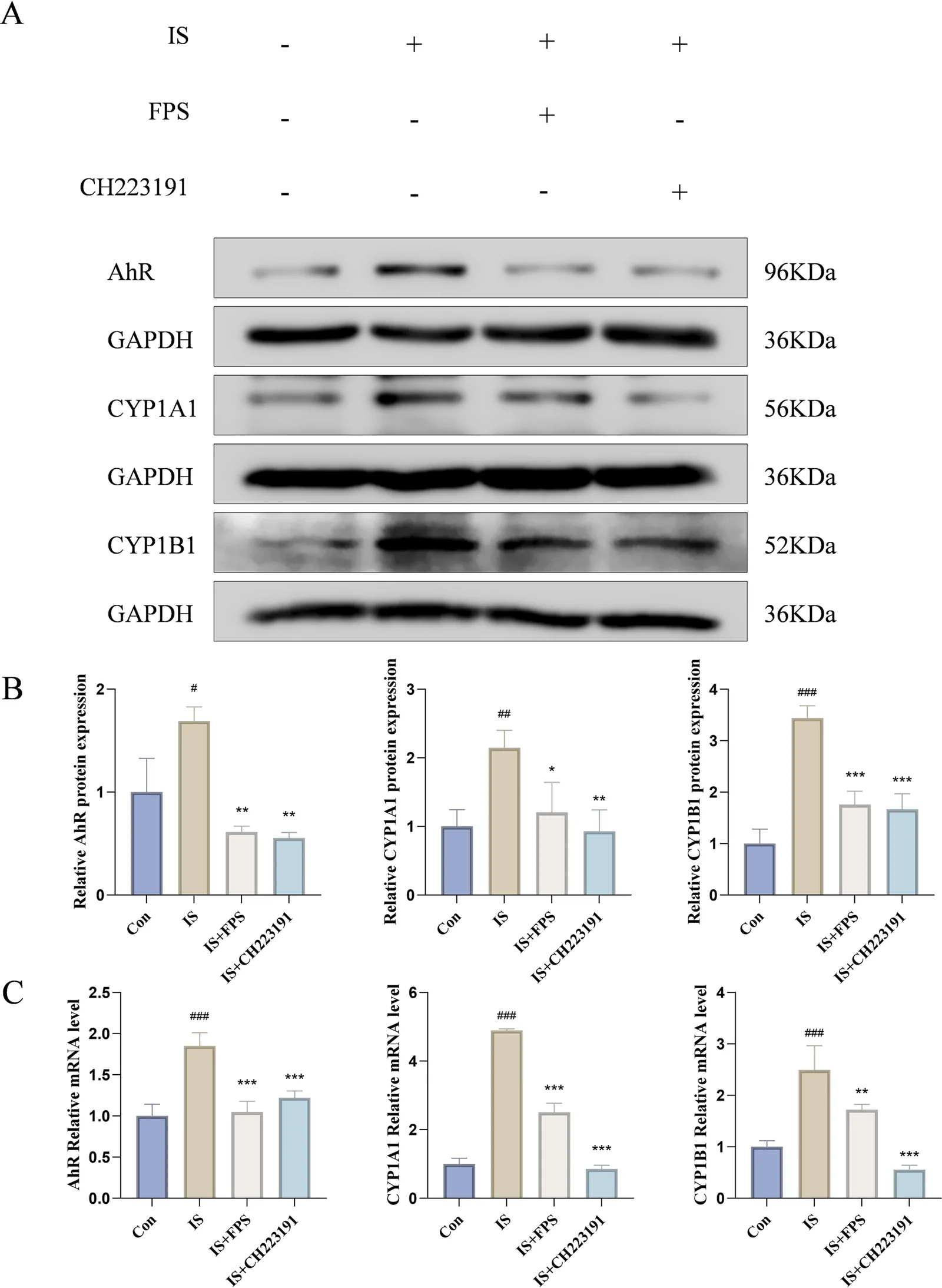
FPS and CH223191 inhibit IS-induced *AhR* pathway activation. **(A)** Representative Western blot bands showing the protein expression of *AhR*, *CYP1A1*, and *CYP1B1* in HK-2 cells treated with IS, FPS, or CH223191. *GAPDH* served as the internal loading control. **(B)** Densitometric analysis of the relative protein expression levels of *AhR*, *CYP1A1*, and *CYP1B1*. **(C)** Relative mRNA expression levels of *AhR*, *CYP1A1*, and *CYP1B1* determined by RT-qPCR. Data are expressed as mean ± SEM. ^#^ *p* < 0.05, ^##^ *p* < 0.01, ^###^ *p* < 0.001 versus the Control group; ^*^ *p* < 0.05, ^**^ *p* < 0.01, ^***^ *p* < 0.001 versus the Model group.

Western blot analysis showed that IS stimulation strongly activated the *AhR* pathway. In comparison with the Control group, IS-treated cells exhibited a pronounced upregulation of *AhR* protein and a marked induction of *CYP1A1* and *CYP1B1* (Figures 6A,B), supporting activation of AhR-associated transcriptional responses in renal tubular cells.

Pharmacological inhibition with CH223191 completely abolished these increases, supporting that the expression of these enzymes is AhR-dependent. Notably, FPS treatment produced an inhibitory effect similar to CH223191, significantly suppressing the protein levels of *AhR*, *CYP1A1*, and *CYP1B1*, which suggests that FPS attenuated IS-induced *AhR* activation.

These results were further supported at the transcriptional level by RT-qPCR (Figure 6C). IS treatment markedly elevated mRNA levels of *AhR*, *CYP1A1*, and *CYP1B1*, and both FPS and CH223191 significantly attenuated these increases.

Together, these data suggest that FPS exerts renoprotective effects through attenuation of IS/AhR signaling.

## 4 Discussion

**FIGURE 7:**
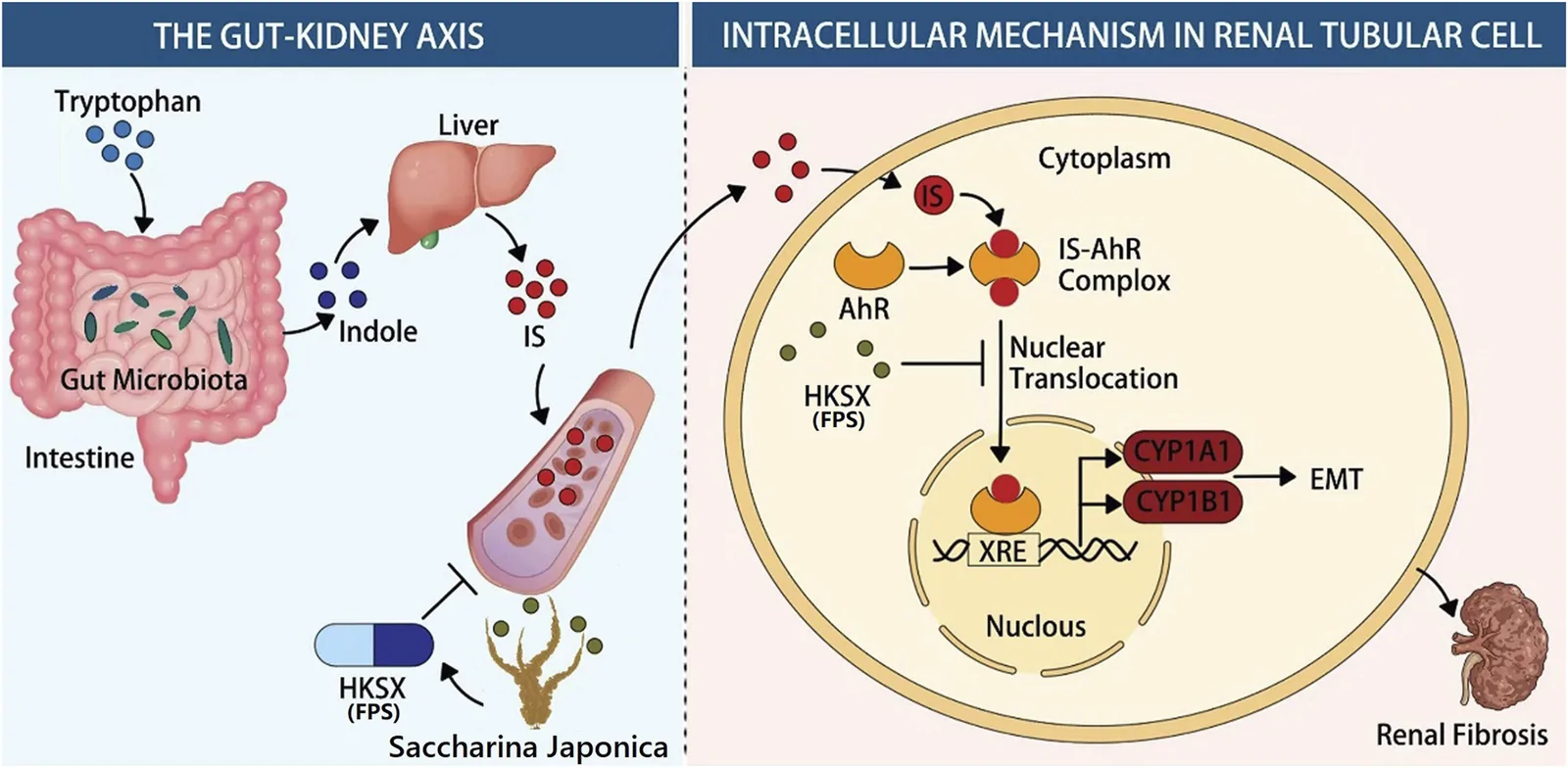
Schematic diagram illustrating the therapeutic mechanism of HKSX and FPS against renal fibrosis via the Gut-Kidney Axis and IS/AhR signaling pathway. (Left Panel) The Gut-Kidney Axis: Dietary tryptophan undergoes metabolism by the gut microbiota to produce indole, which is then sulfated in the liver to form IS. IS enters the circulation and accumulates in CKD. HKSX administration effectively reduces the systemic accumulation of IS. (Right Panel) Intracellular Mechanism in Renal Tubular Cells: Circulating IS enters renal tubular epithelial cells and binds to the *AhR* in the cytoplasm. The IS-AhR complex translocates to the nucleus and binds to Xenobiotic Response Elements (XRE), triggering the transcriptional expression of *CYP1A1* and *CYP1B1*. This activation is associated with EMT-related changes and renal fibrosis progression. FPS, the principal plant-derived metabolite of HKSX, attenuates *AhR* activation by reducing *AhR* nuclear accumulation and is associated with reduced downstream fibrotic signaling.

Renal fibrosis represents a frequent pathological outcome during the progression of CKD to ESRD and underscores the importance of developing effective antifibrotic therapies (Humphreys, 2018; Nastase et al., 2018). In this study, we established an adenine-induced CKD rat model that exhibits renal dysfunction and severe tubulointerstitial fibrosis (Tang et al., 2021), and evaluated the therapeutic potential of a marine-derived drug, HKSX, which is commonly employed for managing CKD in China. In this study, HKSX treatment significantly improved renal function, as shown by dose-dependent reductions in serum creatinine, BUN, and urinary protein levels, along with preserved renal architecture. Notably, the renoprotective effect of high-dose HKSX was comparable to that of AST-120, a standard oral adsorbent. Consistent with the *in vitro* findings, HKSX and its principal plant-derived metabolite, FPS, exhibited anti-fibrotic effects similar to those observed with the *AhR* inhibitor CH223191. Oral fucoidan can be absorbed and preferentially accumulates in the kidney (AUC_0–t_ = 10.74 µg·h/g; Cmax = 1.23 µg/g), providing pharmacokinetic evidence supporting its renal distribution (Pozharitskaya et al., 2018). Importantly, our findings suggest that the antifibrotic effects of HKSX may involve two complementary mechanisms: reduction of systemic IS levels via modulation of the gut–kidney axis and suppression of IS-induced *AhR* signaling in renal tubular cells (Figure 7). However, the relative quantitative contribution of these two mechanisms cannot be determined from the present study and warrants further investigation through dedicated pharmacokinetic and tissue distribution analyses.

Accumulation of gut-derived uremic toxins is a hallmark of disrupted gut-kidney crosstalk in CKD (Tsuji et al., 2024; Wu et al., 2025) and its complications, such as cognitive dysfunction, heart failure (Andrews et al., 2025; Balestrieri et al., 2023; Zhang et al., 2025). Under physiological conditions, dietary tryptophan undergoes metabolism by the gut microbiota to produce indole, which is then sulfated in the liver to form IS and excreted via renal organic anion transporters (OAT1/3) (Lin et al., 2025; Nigam et al., 2015). In uremia, however, gut dysbiosis and impaired renal clearance synergistically promote systemic IS accumulation (Zhang et al., 2025). In this study, adenine-induced model exhibited markedly elevated serum IS levels, suggesting a state of metabolic intoxication. IS acts as a potent endogenous ligand for *AhR*, and its accumulation is associated with activation of pathological signaling cascades involved in renal injury. (Ichii et al., 2014; Lano et al., 2020; Sun and Xia et al., 2025; Xie et al., 2024). HKSX treatment significantly reduced serum IS levels, suggesting that its sulfated polysaccharides may modulate the gut-kidney axis. This is consistent with recent studies suggesting that fucoidan may alleviate renal fibrosis through modulation of the gut microbiota, including regulation of the Akkermansia muciniphila-acetate-NEU1-TLR4-NF-κB axis (Wang et al., 2025), modulation of tryptophan metabolism (Zhang et al., 2026), and protection of the renal endothelial glycocalyx (Xin et al., 2026). Based on these insights, future studies are warranted to further elucidate the role of FPS in modulating gut microbiota composition and function, for example, using 16S rRNA sequencing to profile microbial communities and fecal microbiota transplantation experiments to validate causality in renal protection.

Considering effectiveness and safety, natural products are recently being developed for novel therapeutics in CKD treatment. Fucoidan, a fucose-rich sulfated polysaccharide derived from brown algae (Phaeophyceae), serves as the principal bioactive ingredient of HKSX. It is increasingly recognized for its ability to modulate cellular signaling and tissue remodeling (Feng et al., 2023; John et al., 2025; Senni et al., 2011). Different from the oral IS absorbent AST-120, HKSX and its principal plant-derived metabolite, FPS, were associated with reduced systemic IS accumulation and attenuation of IS-induced AhR-mediated fibrotic signaling in renal tissues. As well researched, FPS has been reported to exert anti-inflammatory, antioxidant, and antifibrotic effects and to regulate multiple signaling pathways, including Nrf2/HO-1, ERK and p38 MAPK, NF-κB, TGF-β1, known to be associated with CKD pathobiology (AlKahtane et al., 2020; Chen et al., 2015; Yu et al., 2020; Zahan et al., 2022). However, limited study of FPS focuses on Gut-Kidney Axis. The Gut-Organ axis is widely recognized as a pivotal mediator of systemic health, primarily through gut-derived immune, metabolic, and inflammatory signaling. FPS have been confirmed to modulate gut microbiota composition, function and restore intestinal barrier integrity (Sun and Li et al., 2025). This comprehensive blocking Gut-Kidney Axis strategy may confer superior protection against CKD fibrosis progression.

Several limitations of this study should be noted. Firstly, the specific impact of HKSX on gut microbiota composition and tryptophan metabolism warrants further characterization via metabolomic approaches. Secondly, although we showed that FPS could inhibit IS/AhR signaling, the precise transcriptional mechanism remains to be further clarified. A limitation of the present study is that direct evidence of IS-induced recruitment of *AhR* to the *CYP1A1/CYP1B1* promoter regions was not obtained. Nevertheless, our functional assays demonstrated that FPS reduced IS-induced *AhR* nuclear accumulation and downstream α-SMA expression, supporting its role in modulating *AhR* signaling. Chromatin immunoprecipitation (ChIP)-based analysis of *AhR* occupancy at the *CYP1A1/CYP1B1* promoters represents an important direction for future investigation. Thirdly, although previous pharmacokinetic studies have demonstrated that orally administered fucoidan can be absorbed and distributed to renal tissue (Pozharitskaya et al., 2018), we did not assess the pharmacokinetics or renal tissue distribution of FPS under the present experimental conditions. Therefore, the relative contributions of the direct renal effects of FPS and its indirect systemic actions require further investigation using dedicated pharmacokinetic and tissue distribution studies. Finally, the translational relevance of these findings requires validation in other CKD models, such as 5/6 nephrectomy, folic acid-induced CKD, cisplatin-induced CKD, and diabetic nephropathy, to confirm broad applicability.

Based on our findings, we suggest that the renoprotective effects of HKSX and FPS are likely mediated by two complementary mechanisms: reduction of systemic IS accumulation through modulation of the gut–kidney axis (supported by *in vivo* data), and attenuation of IS-induced *AhR* signaling in renal tubular cells (supported by *in vitro* data). However, the relative quantitative contribution of these two mechanisms cannot be determined from the present study and warrants further investigation.

In summary, this study highlights the pivotal role of IS/AhR signaling in promoting EMT and renal fibrosis in a CKD model. Our findings suggest that HKSX, via its marine-derived active ingredient Fucoidan (FPS), is associated with potent renoprotection by concurrently reducing systemic IS accumulation and attenuating AhR-mediated fibrotic signaling. These findings support the potential of marine sulfated polysaccharides as promising multi-target candidates for CKD management and offer a scientific rationale for therapeutic strategies targeting the Gut-kidney Axis.

## 5 Conclusion

In summary, this study comprehensively evaluates the renoprotective efficacy and underlying molecular mechanism of the marine-derived formulation HKSX and its principal plant-derived metabolite, FPS, in renal fibrosis. Our findings suggest that HKSX may exert its therapeutic effects through a distinct “Dual-Intervention” strategy targeting the disrupted gut-kidney axis. Firstly, HKSX was associated with modulation of systemic tryptophan metabolism and reduced accumulation of the uremic toxin IS, accompanied by attenuation of profibrotic signaling. Secondly, FPS is associated with attenuation of IS/AhR signaling, reduced *AhR* nuclear accumulation, decreased downstream *CYP1A1* and *CYP1B1* expression, and attenuation of EMT-related changes and renal fibrosis. These findings support the pivotal role of IS/*AhR* signaling in renal fibrogenesis and suggest that marine sulfated polysaccharides may represent promising multi-target candidates for the clinical management of CKD.

## Data Availability

The original contributions presented in the study are included in the article/Supplementary Material, further inquiries can be directed to the corresponding authors.
