# Supplementary material for: Haikunshenxi capsule alleviates renal fibrosis in chronic kidney disease via modulation of indoxyl Sulfate–Aryl hydrocarbon receptor signaling
Source: Front Pharmacol. 2026 Aug 11;17:1819011. doi: 10.3389/fphar.2026.1819011 (PMC13503379; doi:10.3389/fphar.2026.1819011)
Supplement: Supplementary file 1 [file Table1.docx]

Supplementary Material

# 1 Supplementary Figures and Tables

## 1.1 Supplementary Figure

**Supplementary Figure S1.** Effects of FPS on HK-2 cells. (A) CCK-8 cell viability assay showing the effects of various concentrations of IS ( top panel) and FPS ( bottom panel) on HK-2 cell viability. (B) Western blot analysis of α-SMA protein expression in Control, IS, IS+FPS, FPS-only, and IS+CH223191 groups, demonstrating that FPS alone does not significantly alter α-SMA levels. GAPDH was used as a loading control. Data are presented as mean ± SEM (n = 3). Statistical significance: ^*^*p* < 0.05, ^**^*p* < 0.01, ^***^*p* < 0.001 versus Control.

## 1.2 Supplementary Tables
